# Supplementary material for: A Composite Fabric with Dual Functions for High-Performance Water Purification
Source: Materials (Basel). 2022 Aug 26;15(17):5917. doi: 10.3390/ma15175917 (PMC9457210; doi:10.3390/ma15175917)
Supplement: Supplementary file 1 [file materials-15-05917-s001.zip › materials-1835474-supplementary.pdf]

materials

Supporting information

A Composite Fabric with Dual Functions for High-Performance Water Purification

Yankuan Tian [1], Xin Yang [1], Long Xu [1], Xueli Wang [2], Jianyong Yu [2], Dequn Wu [1], Faxue Li [1], Tingting Gao [1,*]

[1] Key Laboratory of Textile Science & Technology, Ministry of Education, College of Textiles, Donghua University, Shanghai 201620, China
[2] Innovation Center for Textile Science & Technology, Donghua University, Shanghai 201620, China
* Correspondence: gaott@dhu.edu.cn

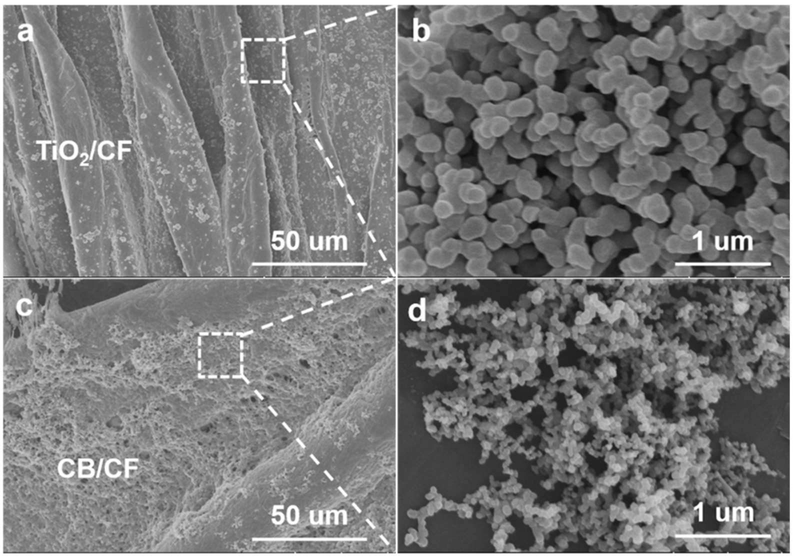

Figure S1. SEM image of (a) and (b) TiO2/CF, (c) and (d) CB/CF.

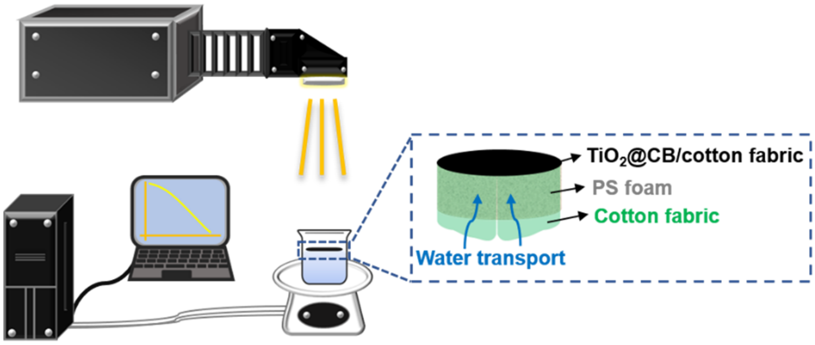

Figure S2. Schematic experimental setup for evaluating solar-vapor generation performance and photocatalytic activity test. The PS foam is covered by the cotton fabric wall.

Owing to the lightweight and low thermal conductivity (<0.04 W m-1 K-1) of polystyrene foam (PS foam), so the PS foam was used as a role for thermal insulation and self-floating. The PS foam can localize the heat in the air-water at the interface and reduce heat loss, achieving an efficient solar-driven evaporation process. The hydrophilic cotton fabric, which was used to wrap around the PS foam, can be used as a channel for transporting water to ensure adequate water supply for the light absorption layer.

Regarding the calculation of evaporation enthalpy in this work, we do not consider the factor of MB, assuming the evaporation enthalpy of MB solution is similar to pure water.

The calculation of efficiency is related to the enthalpy of the liquid-vapor phase, although the 2260 J g-1 had been used as the value of latent heat enthalpy (h_l) by many reported works. The value is dependent on the temperature. And h_l changes under different temperatures, and their relationship can be described by Equation S1[1,2]:

$$h_{lv} = \alpha + \beta T + \gamma T^{1.5} + \delta T^{2.5} + \varepsilon T^3 \quad \text{(S1)}$$

Where α = 2500.304, β= -2.2521025, γ = -0.021465847, δ = 3.1750136×10-4, ε = -2.8607959×10-5 are constants and T is the temperature of pure water. Thus, the h_l of pure water at 33.1, 36.2, 47.2, and 48.7 ℃ were calculated to be 2423.9, 2414.8, 2389.4, and 2384.6 J g-1, respectively.

Due to the interfacial interaction between water and surface groups of materials, the actual liquid-vapor phase change enthalpy (h_s) of water differs from the theoretical value under a specific temperature. It needs to be further determined by measurement. Assuming that the water evaporation is powered by identical energy input (U_{in}), according to the reported method [2], the h_s can be determined using the following Equation:

$$U_{in} = h_w\, m_w = h_s\, m_s$$

Where h_w and h_s are the theoretical liquid-vapor phase change enthalpy of water and actual liquid-vapor phase change enthalpy of water on samples at a specific temperature, respectively. m_w and m_s are the mass change rate of pure water and water on samples under the same condition, respectively.

TG measurements were conducted to record the constant-temperature mass change rate of pure water and water-soaked TiO2@CB/CF. Specifically, 5 μL of pure water and water-soaked TiO2@CB/CF with the same water volume were respectively dropped into the ceramic crucibles in a TG analyzer. The TiO2@CB/CF was cut to the same surface area as pure water. The ceramic crucibles were then transferred into the heating chamber, and the mass change rate was recorded at a constant temperature of 47.2 °C. The mass change rate of pure water and the TiO2@CB/CF was measured to be 0.199 mg min-1 and 0.219 mg min-1. As a result, the actual liquid-vapor phase change enthalpy (hs) of water for the TiO2@CB/CF is calculated to be 2171.2 J g-1. The actual liquid-vapor phase change enthalpy (hs) of water for the cotton fabric, TiO2/CF, and CB/CF-np can be determined using the same test method. The results are displayed in Table S1.

Table S1. h_l of the actual value of different samples.

Samples	Tempeture/℃	h_w/ J g^{-1}	h_s/ J g^{-1}
Cotton fabric	33.1	2423.9	2372.6
TiO_2/CF	36.2	2414.8	2293.3
TiO_2@CB/CF	47.2	2389.4	2171.2
CB/CF	48.7	2384.6	2175.1

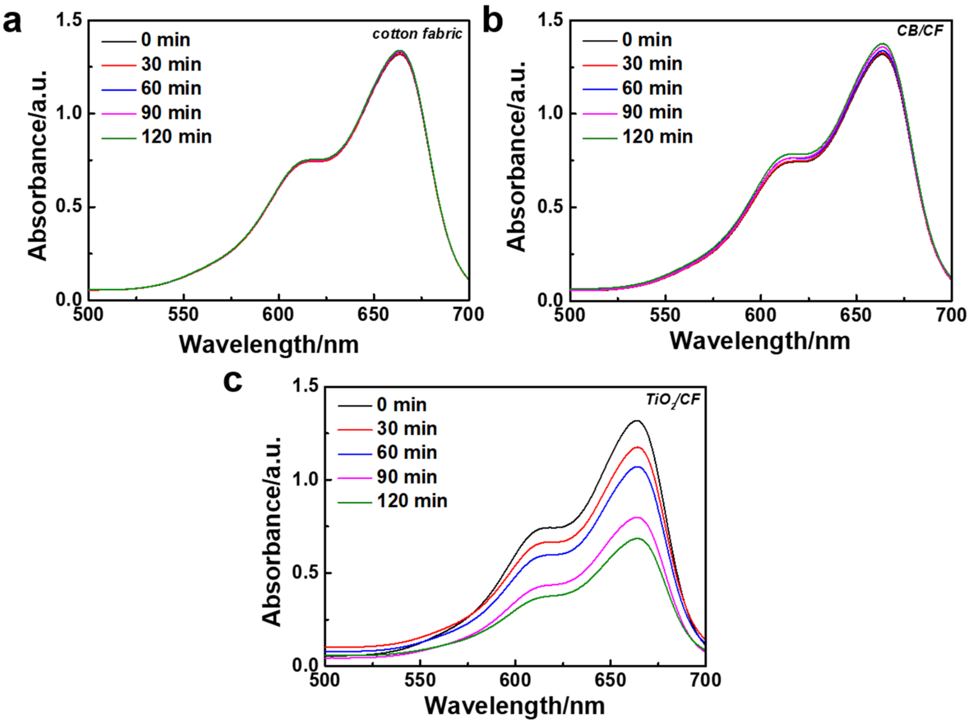

Figure S3. The UV-vis absorption spectra show photodegradation of MB solution with (**a**) cotton fabric, (**b**) CB/CF, and (**c**) TiO_2/CF.

It has been reported that the concentration of a solution can seriously affect the water evaporation rate [8, 9]. With the solution evaporation going on, the concentration of the solution increases, and thus the water evaporation rate decreases.

To further investigate the role of TiO_2 and the effect of MB concentration variation, we prepared different MB concentrations (5, 10, and 20 mg L^{-1}) as the treatment fluid to explore the solar-vapor generation performance of TiO_2@CB/CF and CB/CF.

From Figure S2, we can find that the evaporation rates of the TiO_2@CB/CF are 1.42, 1.28, and 1.05 kg m^{-2} h^{-1} in different MB concentrations (5, 10, and 20 mg L^{-1}), respectively. And the evaporation rates of the CB/CF are 1.37, 1.21, and 0.99 kg m^{-2} h^{-1} in different MB concentrations (5, 10, and 20 mg L^{-1}), respectively.

For the same sample, with the increase of MB concentration, the evaporation rate has a decreasing trend. The reason for this result is that it is difficult for fibers to transport MB solution and solar-driven evaporation when the concentration is too high. In addition, for the MB solution with the same concentration, the evaporation rate of TiO_2@CB/CF is higher than CB/CF. The reason is that TiO_2 can catalyze and degrade MB, thereby reducing the solution concentration.

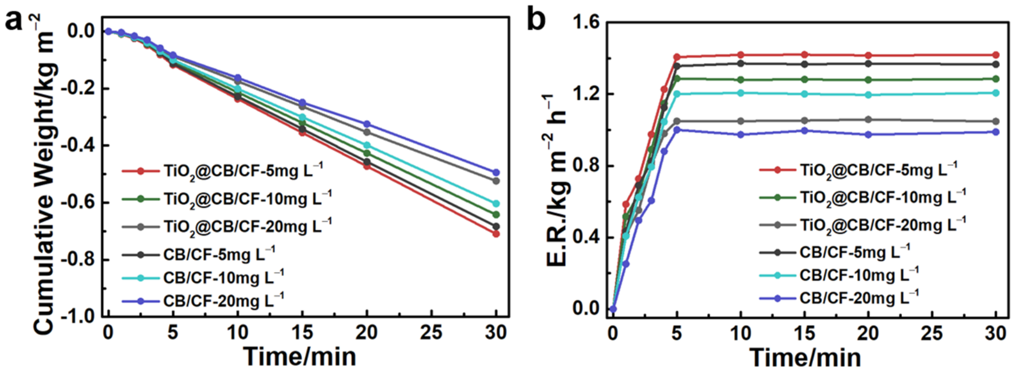

Figure S4. The solar-vapor generation performance of TiO_2@CB/CF and CB/CF in different MB concentrations.

References

1. Liu, Z.; Wu, B.; Zhu, B.; Chen, Z.; Zhu, M.; Liu, X. Continuously Producing Watersteam and Concentrated Brine from Seawater by Hanging Photothermal Fabrics under Sunlight. *Adv. Funct. Mater.* **2019**, *29*, 1905485.
2. Liu, Z.; Zhou, Z.; Wu, N.; Zhang, R.; Zhu, B.; Jin, H.; Zhang, Y.; Zhu, M.; Chen, Z. Hierarchical Photothermal Fabrics with Low Evaporation Enthalpy as Heliotropic Evaporators for Efficient, Continuous, Salt-Free Desalination. *ACS Nano* **2021**, *15*, 13007–13018.
3. Ma, X.; Fang, W.; Guo, Y.; Li, Z.; Chen, D.; Ying, W.; Xu, Z.; Gao, C.; Peng, X. Hierarchical porous SWCNT stringed carbon polyhedrons and PSS threaded MOF bilayer membrane for efficient solar vapor generation. *Small* **2019**, *15*, 1900354.
4. Xu, Y.; Liu, D.; Xiang, H.; Ren, S.; Zhu, Z.; Liu, D.; Xu, H.; Cui, F.; Wang, W. Easily scaled-up photothermal membrane with structure-dependent auto-cleaning feature for high-efficient solar desalination. *J. Membrane Sci.* **2019**, *586*, 222.
5. Wu, S.; Xiong, G.; Yang, H.; Gong, B.; Tian, Y.; Xu, C.; Wang, Y.; Fisher, T.; Yan, J.; Cen, K.; et al. Multifunctional solar waterways: Plasma-enabled self-cleaning nanoarchitectures for energy-efficient desalination. *Adv. Energy Mater.* **2019**, *9*, 1901286.
6. Xu, N.; Hu, X.; Xu, W.; Li, X.; Zhou, L.; Zhu, S.; Zhu, J. Mushrooms as efficient solar steam-generation devices. *Adv. Mater.* **2017**, *29*, 1606762.
7. Li, X.; Xu, W.; Tang, M.; Zhou, L.; Zhu, B.; Zhu, S.; Zhu, J. Graphene oxide-based efficient and scalable solar desalination under one sun with a confined 2D water path. *Proc. Natl. Acad. Sci. USA* **2016**, *113*, 13953.
8. Kuang, Y.; Chen, C.; He, S.; Hitz, E.M.; Wang, Y.; Gan, W.; Mi, R.; H.L.; A high-performance self-regenerating solar evaporator for continuous water desalination. *Adv. Mater. USA* **2019**, *31*, 1900498.
9. Liu, Z.; Song, H.; Ji, D.; Li, C.; Cheney, A.; Liu, Y.; Zhang, N.; Zeng, X.; Chen, B.; Gao, J.; et al. Extremely cost-effective and efficient solar vapor generation under nonconcentrated illumination using thermally isolated black paper. *Glob. Chall.* **2017**, *1*, 1600003.

.
